# *Pasteurella multocida* toxin- induced osteoclastogenesis requires mTOR activation

**DOI:** 10.1186/s12964-015-0117-7

**Published:** 2015-09-14

**Authors:** Bianca Kloos, Sushmita Chakraborty, Sonja G. Lindner, Katrin Noack, Ulrike Harre, Georg Schett, Oliver H. Krämer, Katharina F. Kubatzky

**Affiliations:** Zentrum für Infektiologie, Medizinische Mikrobiologie und Hygiene, Universitätsklinikum Heidelberg, Im Neuenheimer Feld 324, 69120 Heidelberg, Germany; Center for Molecular Biomedicine (CMB), Department of Biochemistry, University of Jena, Hans Knöll Str. 2, 07745 Jena, Germany; Integrated Research and Treatment Center, Center for Sepsis Control and Care (CSCC), Jena University Hospital, Erlanger Allee 101, 07747 Jena, Germany; Department of Internal Medicine 3 and Institute of Clinical Immunology, University of Erlangen-Nuremberg, 91054 Erlangen, Germany; Institute of Toxicology, Medical Center of the University Mainz, Obere Zahlbacher Str. 67, 55131 Mainz, Germany

## Abstract

**Background:**

*Pasteurella multocida* toxin (PMT) is a potent inducer of osteoclast formation. Pigs suffering from an infection with toxigenic *Pasteurella multocida* strains develop atrophic rhinitis characterised by a loss of turbinate bones and conchae. However, on the molecular level the process of bone loss remains largely uncharacterised.

**Results:**

Recently it was found that PMT activates the serine/threonine kinase mammalian target of rapamycin (mTOR) in fibroblasts. Using RAW264.7 macrophages, we investigated the role of the mTOR complex 1 (mTORC1) in PMT-mediated osteoclast formation. PMT induces the differentiation of RAW264.7 macrophages into multinucleated, tartrate resistant acid phosphatase (TRAP) positive osteoclasts that are capable to resorb bone. In the presence of the mTORC1 inhibitor rapamycin, PMT was significantly less able to induce the formation of TRAP-positive osteoclasts. Accordingly, the resulting resorption of bone was strongly reduced. A major target of mTOR is the 70 kDa ribosomal protein S6 kinase 1 (p70 S6K1). Activated p70 S6K1 decreases the expression of programmed cell death protein 4 (PDCD4), a negative transcriptional regulator of osteoclastogenesis, at the protein and gene level. Ultimately this results in the activation of c-Jun, a component of the activator protein 1 (AP-1) complex, which is a major transcription factor for the induction of osteoclast-specific genes. We now demonstrate that c-Jun and its downstream target, the osteoclast-specific bone degrading protease cathepsin K, are upregulated upon PMT treatment in an mTOR-dependent manner.

**Conclusions:**

Activation of mTOR signalling plays a central role in the formation of osteoclasts through the bacterial toxin PMT. On the molecular level, PMT-induced activation of mTOR leads to down regulation of PDCD4, a known repressor of AP-1 complex, culminating in the activation of c-Jun, an essential transcription factor for triggering osteoclastogenesis.

**Electronic supplementary material:**

The online version of this article (doi:10.1186/s12964-015-0117-7) contains supplementary material, which is available to authorized users.

## Background

It is well known that PMT is the causative factor of porcine atrophic rhinitis. This disease is characterised by increased osteoclastogenesis and osteoclast activity as well as an inhibition of osteoblast function, eventually leading to the degradation of bone [1]. Despite the economic impact of this disease due to reduced growth rates of livestock [2], the molecular mechanisms activated by PMT are just starting to become unravelled [3]. PMT acts as a deamidase for the Gα subunits of heterotrimeric G proteins [4]. As a consequence, a glutamine residue is changed into a glutamic acid residue, thereby inhibiting the intrinsic GTPase activity of the Gα subunit and rendering it constitutively active [5]. Recently it has been published that PMT activates mTORC1 in fibroblasts [6, 7]. The serine/threonine kinase mTOR is a central signalling molecule that connects the activity of a cell to environmental requirements by transducing the extracellular signal into a change in protein translation. Due to this pivotal role in cellular maintenance, abnormal mTOR regulation is often seen in pathologic conditions, including cancer [8]. The activation of mTORC1 can be inhibited by the anti-fungal macrolide rapamycin, which originally led to the name mTOR, for mammalian or mechanistic target of rapamycin. This kinase belongs to the family of PI3K-related kinases and its two best-characterised downstream targets are 4E binding protein-1 (4E-BP1) and p70 S6K1 [9]. The mTOR molecule can interact with different complex partners to create the so-called mTORC1 complex, which is rapamycin sensitive and mTORC2, which is mostly insensitive to rapamycin, respectively. Interaction partners of mTORC1 are the proteins Raptor, proline-rich Akt substrate (PRAS40), mammalian lethal with SEC13 protein 8 (mLST8) and DEP domain-containing mTOR-interacting protein (Deptor), where PRAS40 and Deptor act as negative regulators for mTOR activity [9]. Phosphorylation of PRAS40 and Deptor through Akt or mTOR releases them from the complex and allows signalling [10, 11].

Many clinical data suggest that the inhibition of mTOR signalling decreases bone erosion in diseases such as rheumatoid arthritis, multiple myeloma or neurofibromatosis [12, 13]. Investigations of mTOR signal transduction pathways also suggest that mTOR plays a central role in osteoclastogenesis, since signalling cascades initiated by macrophage colony-stimulating factor (MCSF), receptor activator of nuclear factor kappa-B ligand (RANKL) or tumour necrosis factor (TNF)-α converge downstream on mTOR [14]. As PMT is a potent inducer of osteoclast differentiation, we investigated the effect of mTOR activation in a murine macrophage cell line (RAW264.7 cells) that can be differentiated into osteoclasts [15, 16]. Our studies reveal a central role for mTOR in PMT-driven osteoclast formation. In contrast to recent studies where it was shown that PMT-mediated mTOR activation influences the proliferation of Swiss3T3 cells through an autocrine pathway involving the production of connective tissue growth factor (CTGF) [6], we did not find mTOR to be involved in cellular proliferation of RAW264.7 cells nor in the production of cytokines. However, PMT-induced formation of functional osteoclasts was reduced in the presence of the mTORC1 inhibitor rapamycin as shown by the quantification of TRAP-positive multinucleated cells, activity of the cysteine protease cathepsin K and the ability to resorb bone. The mTOR-dependent signalling pathway involves the activation of the direct mTOR target kinase p70 S6K1 which ultimately allows the phosphorylation and subsequent degradation of PDCD4 [17, 18]. Loss of PDCD4 releases the c-Jun component of the AP-1 transcription factor complex [19]. Through mTORC1-dependent c-Jun phosphorylation, osteoclastic genes such as the bone degrading protease cathepsin K are translated.

## Results

**Fig. 1:**
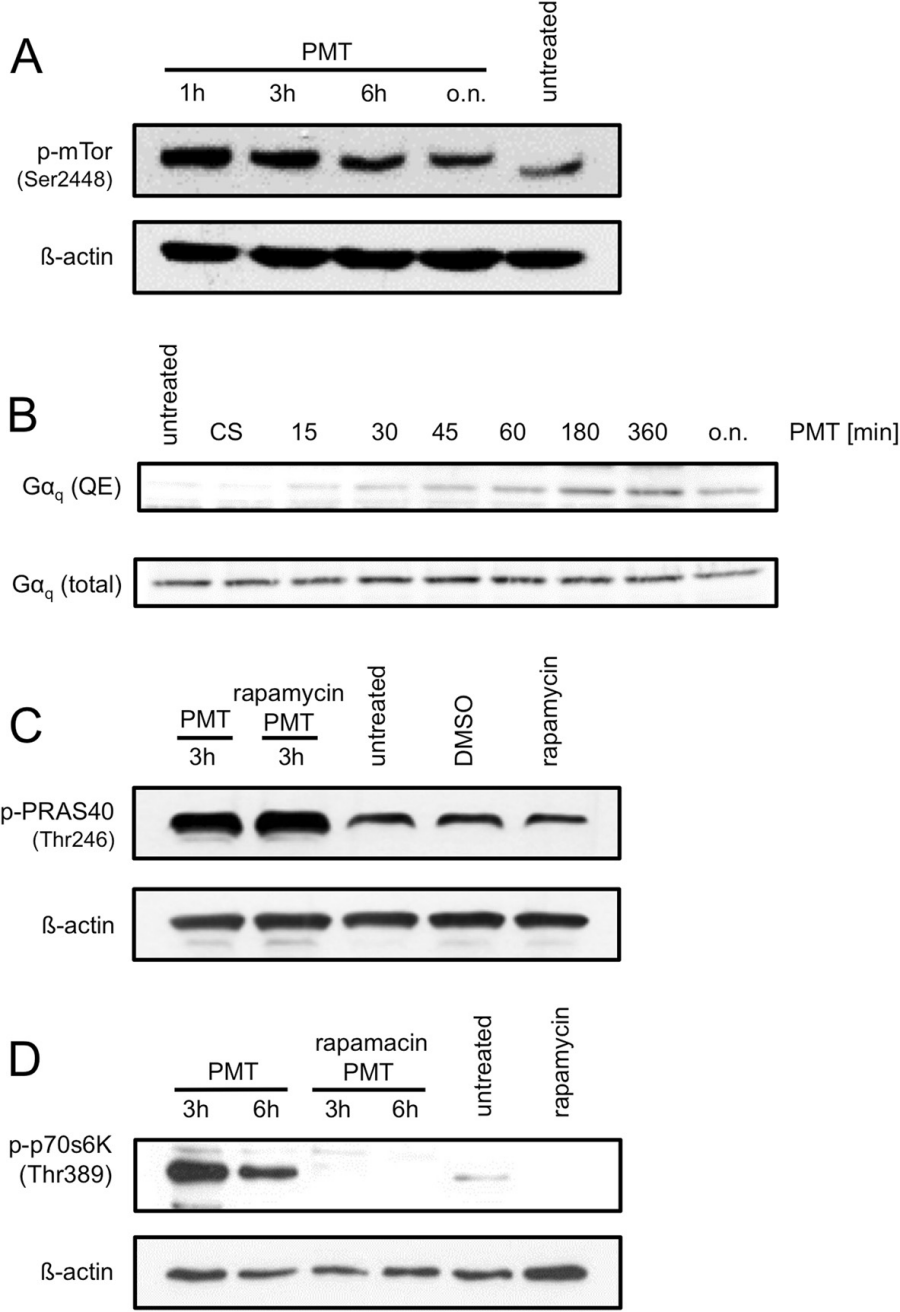
The mTOR pathway is activated in response to PMT. RAW264.7 cells were stimulated with PMT (5 nM) w or w/o rapamycin (10 ng/ml) for the indicated period of time or were left unstimulated, treated with the solvent control DMSO or rapamycin only. Cells were lysed and the proteins immunoblotted. (**a**) mTOR Ser2448 phosphorylation was determined using a specific antibody and β-actin was used as loading control. (**b**) To investigate the uptake and intracellular action of PMT, cells were stimulated for the indicated time-points with 5 nM PMT and lysed. As a control, cells were stimulated with a catalytically inactive mutant of PMT (PMT^C1165S^). The immunoblot was probed with a specific antibody detecting the deamidated form of Gα_q_ (Q209E) or total Gα_q_. (**c**, **d**) mTOR-dependent protein phosphorylation from RAW264.7 cells stimulated with PMT was detected using specific antibodies for PRAS40 (Thr246) or p70 S6K1 (Thr389); ß-actin was used as a loading control. The results shown are one representative example of three independent experiments

In order to see whether mTOR activation through PMT plays a role in immune cells, we stimulated RAW264.7 cells with PMT and checked for mTOR phosphorylation. Indeed we found that mTOR was activated by treatment with PMT (Fig. 1a). Interestingly, mTOR phosphorylation was rapidly induced (1 h) and lasted, due to the constitutive G protein activation that is a characteristic feature of PMT signalling [20], for at least 24 h.

Since we observed PMT-induced mTOR phosphorylation quite early, we verified that the observed mTOR activation occurred indeed as a consequence of intracellular PMT signalling. We therefore investigated the kinetics of PMT-induced intracellular deamidation of the Gα_q_ subunit by a Gα_q_ antibody, specifically recognising the Q209E modification that is generated by the targeting and subsequent deamidation through PMT [21]. We found that the uptake of PMT must be much faster than previously suggested as intracellular signalling events can be detected as early as 15–30 min after stimulation (Fig. 1b).

Phosphorylation of PRAS40 on Thr246 regulates mTORC1 activity by rendering PRAS40 to prefer binding to 14-3-3 rather than to mTORC1, for which it is an inhibitor of kinase activity. When cells were treated with PMT for 3 h, PRAS40 was robustly phosphorylated on Thr246. However, PMT-induced phosphorylation of PRAS40 was unaffected by rapamycin treatment, indicating that PMT-induced PRAS40 phosphorylation is mTOR independent (Fig. 1c).

Next we investigated the activation of the major mTOR substrate, the Ser/Thr kinase p70 S6K1 which itself is important for the phosphorylation of the ribosomal protein S6 involved in mRNA translation. Western blot analysis for p70 S6K1 phosphorylation on threonine residue 389, which is critical for activation of its kinase activity, showed that the protein is highly phosphorylated in cells incubated with PMT. As expected, this phosphorylation was completely abrogated in the presence of the mTORC1 inhibitor rapamycin (Fig. 1d).

**Fig. 2:**
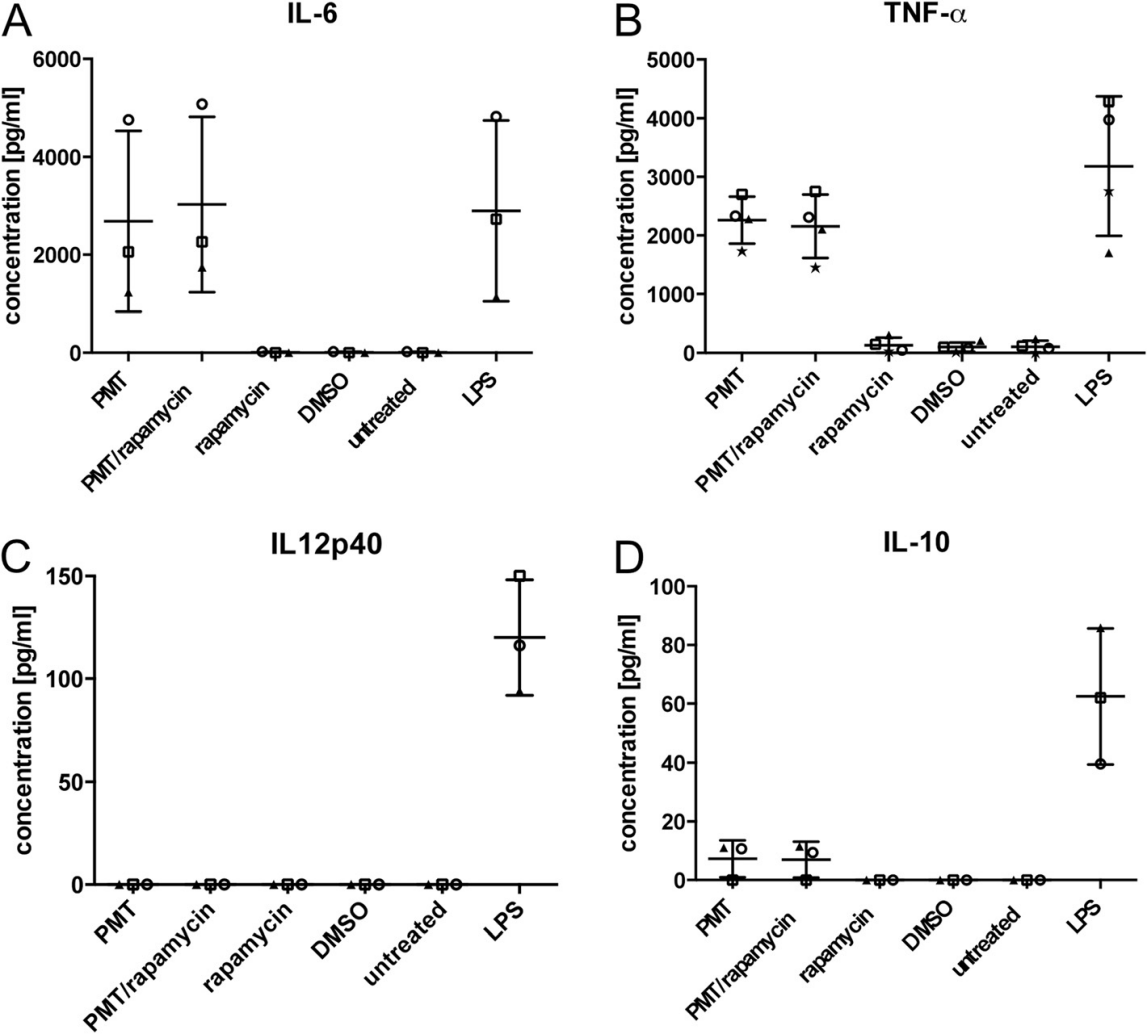
PMT stimulated release of TNF-α or IL-6 is independent of the mTOR pathway. RAW264.7 cells were stimulated with PMT (5 nM) w or w/o pre-incubation with rapamycin (10 ng/ml) for 1 h. As control, cells were untreated, treated with the solvent control DMSO, with rapamycin or LPS (100 ng/ml) as positive control. After 24 h, supernatants were removed and subjected to ELISA for the measurement of (**a**) IL-6-, (**b**) TNF-α-, (**c**) IL12p40- or (**d**) IL-10 release. Shown are the results of three independent experiments (mean ± SD; *n* = 3)

As mTOR has been reported to limit the production of inflammatory cytokines in immune cells, we checked the effect of rapamycin on PMT induced cytokines in RAW264.7 cells using ELISA (Fig. 2a-d). We determined the secretion of pro-inflammatory cytokines such as IL-6, TNF-α or IL12-p40 and the anti-inflammatory cytokine IL-10, respectively, in response to PMT treatment. As a positive control for cytokine induction, the toll-like receptor 4 (TLR4) ligand lipopolysaccharide (LPS) was used. PMT induced the production of IL-6 and TNF-α as reported previously [22, 23], but incubation of the cells with rapamycin did not alter the effect of PMT on the level of cytokine induction. IL12-p40 was not induced by PMT and inhibition of mTOR did not induce IL12-p40 production from these cells. Similarly, the anti-inflammatory cytokine IL-10 was not induced by PMT and rapamycin treatment did not have any effect on IL-10 production. These observations suggest that mTOR does not play a role in the modulation of PMT-mediated cytokine production in RAW264.7 macrophages. This is supported by our previous results that showed that PMT-induced activation of the NF-κB pathway plays a pivotal role in the induction of pro-inflammatory cytokines via RhoA/ROCK activation [24], which is independent of mTOR.

**Fig. 3:**
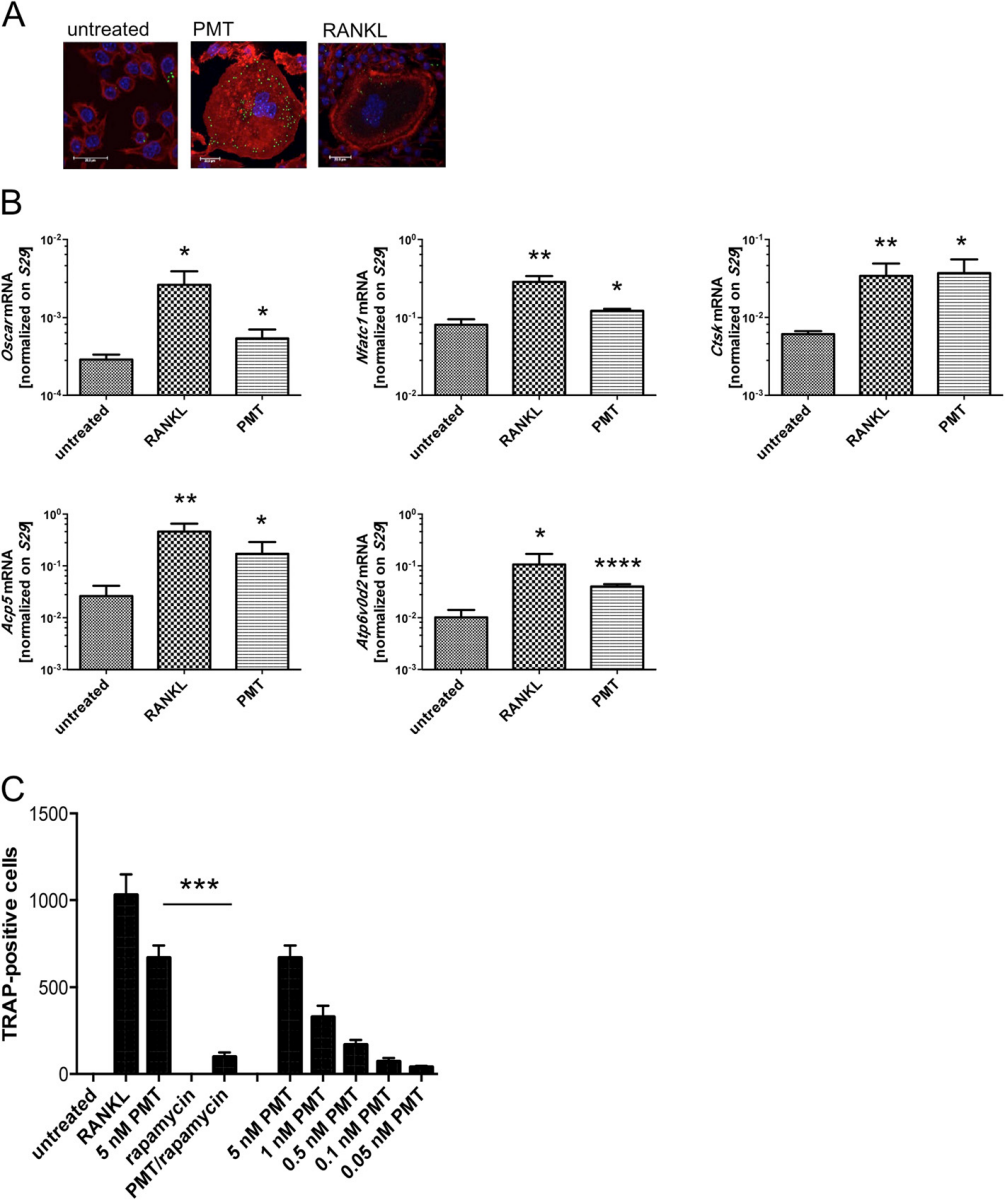
Osteoclast differentiation of RAW264.7 cells with PMT requires mTOR activation and shows a distinct morphology. (**a**) RAW264.7 cells were stimulated with PMT (5 nM) or RANKL (50 ng/ml) as a positive control for osteoclast differentiation. After 4 days of stimulation, cells were labelled in an enzymatic reaction for the activity of the enzyme TRAP (ELF97) in green. Nuclei were stained with DAPI (blue) and the cytoskeleton with TRITC-Phalloidin (red). Pictures were taken by confocal laser scanning microscopy and representative pictures are shown (number of independent experiments *n* ≥3). (**b**) RAW264.7 cells were seeded in 6-well plates, stimulated with 5 nM PMT or 50 ng/ml RANKL for 24 h-48 h before preparation of cDNAs. Quantitative RT-PCR was performed and the graphs display the relative expression for *Oscar*, *Nfatc1*, *Ctsk* (cathepsin K), *Acp5* (TRAP) and *ATP6v0d2* normalized to *S29* expression. The indicated standard deviation was obtained from four experiments (mean ± SD; *n* = 4 and *n* = 3 for *Nfatc1*). Statistical analysis was performed using an unpaired Student’s *t*-test comparing gene expression to the untreated sample (*: *p* ≤ 0.05; **: *p* ≤ 0.005; ***:*p* ≤ 0.0005). (**c**) The numbers of conventionally TRAP-stained cells were quantified after 3 days of stimulation with the indicated concentrations of PMT or 50 ng/ml RANKL in the presence or absence of rapamycin (10 ng/ml). The graph displays the number of TRAP-positive, multinucleated (≥3) cells. The indicated standard deviation was calculated from three independent experiments (mean ± SD; *n* = 3). Statistical analysis was performed using an unpaired Student’s *t*-test (***: *p* ≤ 0.0005)

As RAW264.7 macrophages are capable of differentiating into osteoclasts [15], we investigated the ability of PMT to trigger osteoclastogenesis in these cells. Figure 3a shows that PMT-treated cells have a very distinct morphology with a strong activation of cytoskeletal rearrangement, but with no clearly defined actin ring being detectable. This is presumably caused by the strong activation of the small GTPase RhoA by PMT [24, 25] which is known to cause the formation of stress fibres as opposed to the activation of the related GTPase Rac1 that is needed for the formation of an actin ring [26]. To verify that despite the changed morphology these cells are genuine osteoclasts, we performed RT-PCR analysis of typical osteoclast genes. Figure 3b shows that the genes encoding for osteoclast associated receptor (OSCAR), nuclear factor of activated T cells (NFATc1), Cathepsin K, TRAP and the d2 isoform of vacuolar (H^+^) ATPase (v-ATPase) V_o_ domain (ATP6v0d2) were upregulated by treatment with RANKL or PMT, respectively. Although there are differences in the level of gene induction between the two stimuli, the data suggest that PMT treatment induces differentiation towards the osteoclast lineage. To investigate this in more detail, we stimulated RAW264.7 cells with PMT or soluble RANKL and incubated the cells for 3 days to allow for cell differentiation. PMT-treated cells were also grown in the presence of rapamycin, to check for mTOR-mediated effects. Osteoclast differentiation was then evaluated by microscopy checking for the expression of the osteoclast specific phosphatase TRAP and the presence of multiple nuclei (≥3). Figure 3c shows that PMT treatment induced osteoclast differentiation in a concentration-dependent manner. Inhibition of mTORC1 signalling in PMT-treated cells by rapamycin reduced the osteoclast formation significantly, even at the highest concentration of PMT. We therefore conclude that a crucial function of PMT-mediated mTOR activation in macrophages is to support osteoclast differentiation.

**Fig. 4:**
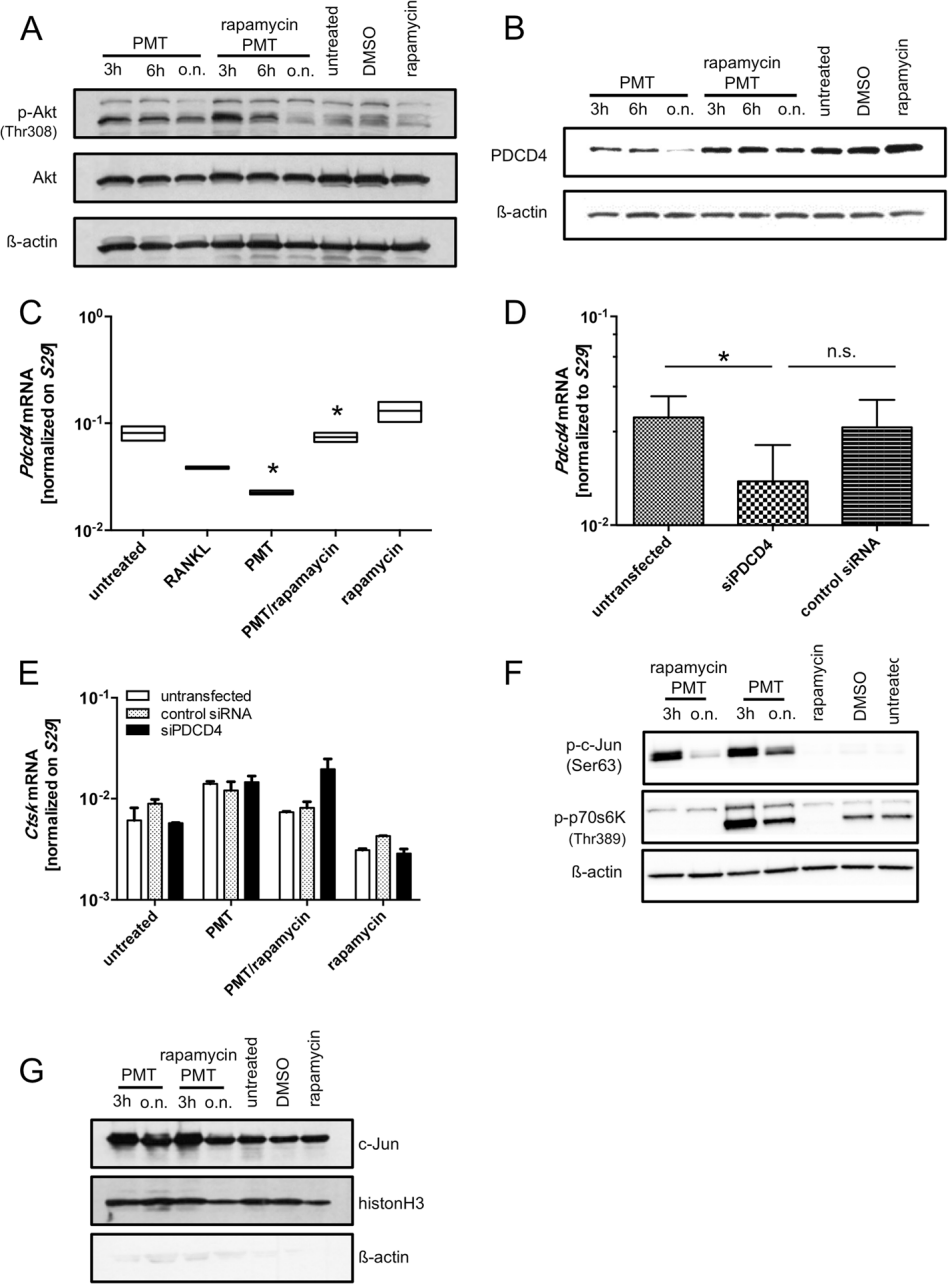
PMT activates targets known to be important for osteoclast differentiation. RAW264.7 cells were left untreated or stimulated with PMT (5 nM) w or w/o rapamycin (10 ng/ml). After incubation for the indicated period of times, cells were examined for Akt (Thr308) phosphorylation or the total amount of Akt (**a**) and PDCD4 (**b**). Shown are representative data from three independent experiments. (**c**) *Pdcd4* expression (mean ± SD; *n* = 2) was determined by RT-PCR in the presence or absence of rapamycin (10 ng/ml) using cells treated with 5 nM PMT or 50 ng/ml RANKL for 6 h (*n* = 2). (**d**) PDCD4 siRNA transfection was performed according to the manufacturer’s protocol. RT-PCR of *Pdcd4* expression was determined after 36 h and was normalized to *S29* expression (*n* = 3). Statistical analysis was performed using a paired Student’s *t*-test (*: *p* ≤ 0.05). (e) 24 h after siRNA transfection, cells were stimulated for 12 h with PMT (5 nM) in the presence or absence of 10 ng/ml rapamycin. The expression of *Ctsk* was monitored by RT-PCR (mean ± SD; *n* = 2). Analysis for (**d**) and (**e**) was performed using the same samples of cDNA (*: *p* ≤ 0.05). (**f **) Phosphorylation of c-Jun (Ser63) and nuclear localisation of c-Jun (**g**) was detected by western blot analysis. Shown are representative data from three independent experiments

To elucidate the signalling pathways of mTOR in PMT-treated macrophages, we had a look at the major activation pathway for mTOR, which is the activation of the PI3 kinase/Akt pathway. In contrast to other PMT-induced signalling pathways, Akt is activated through the Gβγ subunit that is released from the activated G protein, but not through the deamidated Gα subunit itself [27]. Figure 4a shows that PMT induces the activation of Akt by phosphorylation on residue Thr308 in RAW264.7 cells and that this activation is sustained till overnight exposure to PMT. Interestingly, we observed that PMT-induced Akt activation lies upstream of the mTOR complex at early time-points (3 h, 6 h) since treatment with rapamycin did not abrogate Akt phosphorylation. However, after prolonged rapamycin treatment (o.n. stimulation) the phosphorylation of Akt decreases (Fig. 4a). This suggests a potential involvement of mTORC2, which has Akt as a downstream target, and is known to get rapamycin-sensitive after prolonged rapamycin treatment [28].

The heterodimeric transcription factor AP-1 is composed of subunits from the c-Jun and c-Fos families and plays a central role for the induction of osteoclastic genes in co-operation with NFATc1. The activity of AP-1 can be suppressed by PDCD4, which in turn is negatively regulated by activation of the mTOR substrate p70 S6K1 [17, 29]. Decreased PDCD4 levels allow the activation of AP-1, through release of c-Jun molecules from their repressor PDCD4. This subsequently leads to c-Jun phosphorylation and activation by JNK [19] which ultimately results in the release of the suppression of osteoclastic genes. Untreated RAW264.7 cells show a robust expression of PDCD4 that can be further enhanced by treatment with rapamycin (Fig. 4b). On the other hand, stimulation of the cells with PMT for 3 to 6 h strongly decreased the levels of the negative regulator PDCD4 and prolonged treatment with PMT (o.n) resulted in the complete loss of PDCD4 expression. Apart from the regulation of PDCD4 protein levels by mTOR, *Pdcd4* transcription can also be negatively affected by this kinase [30, 31]. When we performed RT-PCR analysis of *Pdcd4* expression, we found that PMT treatment leads to a significant reduction in gene expression that can be reversed by rapamycin treatment (Fig. 4c). Treatment with RANKL also reduced *Pdcd4* expression, however, theses changes were statistically not significant. Next, we investigated the effect of *Pdcd4* expression on the activation of downstream genes. Cathepsin K was described to be an AP-1-dependent gene in RAW264.7 cells [32]. Since *Ctsk* was strongly induced after treatment with PMT (Fig. 3b), we used this gene as a read-out in *Pdcd4* knock-down experiments. RT-PCR analysis confirmed a siRNA-mediated reduction by app. 50% of *Pdcd4* expression compared to untreated cells or control siRNA-treated cells (Fig. 4d). This was corroborated by western blot analysis (Additional file 1: Figure S1). The downregulation of *Pdcd4* in the absence of a signal triggering AP-1 activation does not induce the transcription of downstream genes, however, PMT treatment induced the expression of *Ctsk* in all conditions. This effect was rapamycin-sensitive only in the sets of control cells, whereas the *Pdcd4* knock-down rendered the *Ctsk* expression independent of mTOR (Fig. 4e).

When we investigated c-Jun activation (Fig. 4f), we found that PMT stimulation rapidly induced c-Jun phosphorylation (3 h). However, in contrast to p70s6K activation, only the prolonged stimulation with PMT (o.n.) was mTOR-dependent, while c-Jun activation observed at earlier time-points occurred independently of mTOR. As expected, PMT treatment also led to the rapid accumulation of c-Jun molecules in the nucleus (Fig. 4g). Again, the translocation of c-Jun to the nucleus was only affected after prolonged treatment with rapamycin.

**Fig. 5:**
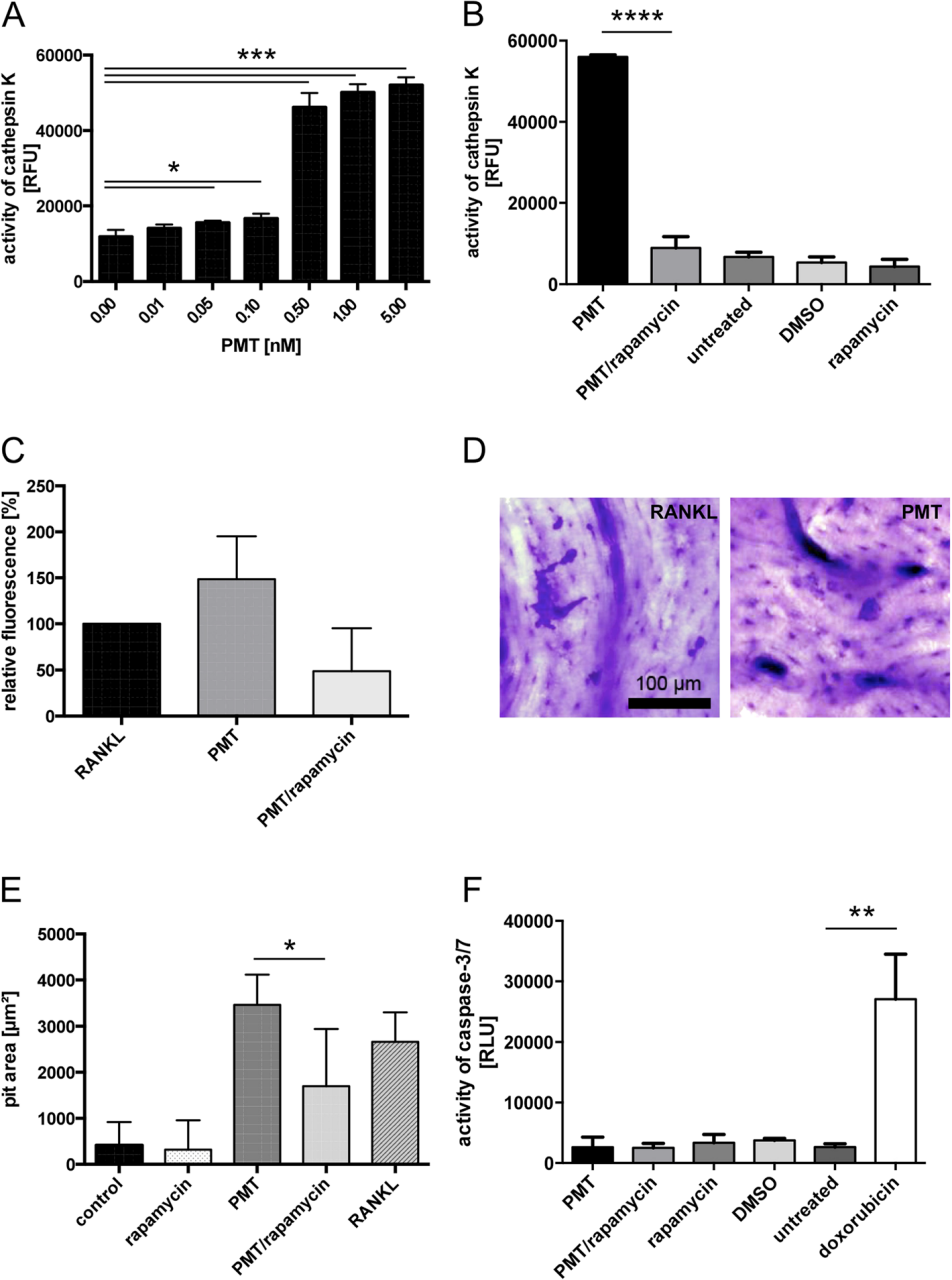
PMT-stimulated mTOR activation is required for the full functional activity of the osteoclasts. (**a**) Lysates of cells stimulated for 4 days with PMT concentrations ranging from 10 pM to 5 nM were analyzed for activity of cathepsin K (cleavage of its specific substrate) by fluorescence detection. (**b**) RAW264.7 cells stimulated with PMT (5 nM) w or w/o rapamycin (10 ng/ml) were analyzed for cathepsin K activity by fluorescence detection. As controls, cells were treated with rapamycin, the solvent control DMSO or were left untreated. (**c**) Cells were plated and stimulated on fluoresceinated calcium phosphate plates for 6 days to allow osteoclast differentiation. A fluorescent dye released into the supernatant dependent on the degree of bone resorption was measured with a fluorometer. (**d **) Osteoclasts were differentiated with RANKL and PMT, respectively, on bone slices and the pit area of resorbed bone was determined. (**e**) Bone resorption induced by RANKL, PMT or PMT in the presence of rapamycin was quantified by determining the pit area. (**f**) Cellular apoptosis was determined by performing a caspase-Glo 3/7 Assay. Cells were incubated for 48 h with PMT (5 nM), the solvent control DMSO, rapamycin (10 ng/ml) or left untreated. As a positive control for apoptosis, cells were incubated with 1 μM doxorubicin for 6 h. Cells were lysed and the luminescence signal was recorded. The indicated standard deviations of all figures were calculated from three independent experiments (mean ± SD; *n* = 3). Statistical analysis for all graphs was performed by unpaired Student’s *t*-test (*: *p* ≤ 0.05, **: *p* ≤0.005, ***: *p* ≤ 0.0005****: *p* ≤ 0.0001)

To investigate the effects of rapamaycin on the functional activity of osteoclasts, we also determined the activity of cellular cathepsin K protein using a reporter assay that releases a fluorescent dye from a sample probe when cleaved by active cathepsin K (Fig. 5a). Cathepsin K was highly induced at PMT concentrations ranging from 0.5 to 5 nM PMT. However, concentrations as low as 0.05 nM were enough to allow detectable production of cathepsin K (Fig. 5a). As our findings indicate that the activation of AP-1 occurs mTORC1 dependently, we hypothesized that treatment with rapamycin would inhibit cathepsin K activity. Indeed, we found cathepsin K activity to be completely abrogated in the presence of rapamycin (Fig. 5b).

Next we tested the degradation of bone matrix using a fluorescent bone resorption assay, where the release of a fluorescent dye into the cell culture supernatant is used to quantify the resorptive activity of osteoclasts on fluoresceinated calcium phosphate plates (Fig. 5c). Here, we observed a decrease in PMT-mediated bone-resorption in the presence of rapamycin due to a decrease in functional osteoclasts (Fig. 3c). Bone resorption could also be detected using bovine bone slices and subsequent toluidine staining. We observed that the bone resorption by PMT-induced osteoclasts was comparable to RANKL-differentiated osteoclasts (Fig. 5d). When osteoclasts were cultivated on bone slices in the presence or absence of rapamycin, a significant reduction of the resorbed pit area was observed in the presence of rapamycin (Fig. 5e). To make sure that the reduced activity of rapamycin-treated osteoclasts is not due to an increase in the number of apoptotic cells, we performed a caspase3/7-Glo assay. The data prove that rapamycin treatment does not lead to an increase in apoptosis (Fig. 5f).

## Discussion

PMT-expressing toxigenic bacterial strains are known to cause bone degradation in pigs. However, it is currently unclear how PMT drives this process and how the bacteria might benefit from the increased osteoclast differentiation. As it was recently shown that PMT is able to activate mTORC1 in a fibroblast cell line [6, 7], we investigated if mTOR activation is important for the differentiation of macrophages into osteoclasts. We found that stimulation with PMT results in osteoclast differentiation in an mTOR-dependent manner in RAW264.7 macrophages. This supports clinical data using the mTOR inhibitor rapamycin as well as data investigating mTOR related signalling pathways after cytokine-mediated osteoclast differentiation that suggest that mTOR is an important factor in bone degradation.

In immune cells activation of the mTOR pathway was described to be part of the response to stimulation by conserved microbial patterns which shifts the cytokine balance towards a diminished release of certain pro-inflammatory cytokines such as IL-6, TNF-α and IL12-p40 and the increased secretion of the anti-inflammatory cytokine IL-10 [33–35]. Analysis whether similar effects on cytokine secretion play a role in PMT-induced activation of the mTOR pathway revealed that the PMT-induced secretion of IL-6 and TNF-α occurs independently of the mTOR pathway (Fig. 2). The pro-inflammatory cytokines IL-6 and TNF-α are known to have a synergistic effect on the activation of the transcription factor AP-1, which was shown to be crucial for osteoclast differentiation [36]. In addition, TNF-α and IL-6 are potent activators of mTOR [14]. It is therefore likely that the observed osteoclast differentiation is a mixture of direct, PMT-mediated signalling events that are related to G protein activation and signal transduction pathways related to the PMT-induced secretion of osteoclastogenic cytokines. Further work will be necessary to delineate the influence of cytokines on PMT-induced osteoclast formation in detail.

We propose the mTOR-dependent removal of the repressor PDCD4 as a central switch to allow osteoclast differentiation by turning on the transcription of osteoclast specific genes through AP-1. PDCD4 activity can be controlled by regulating its stability. Cellular degradation of PDCD4 was shown to be induced by phosphorylation in mTOR-related signalling pathways through p70 S6K1 or Akt, respectively. This phosphorylation enhances binding of the E3-ubiquitin ligase ß-TrCP1 and accelerates the proteasomal degradation of PDCD4 [17, 18]. Here we show that PDCD4 is downregulated after PMT stimulation and that this is indeed dependent on mTOR activation, since treatment of the cells with rapamycin stabilized PDCD4 expression. It is possible that this process is additionally sustained by the secretion of the PMT-induced pro-inflammatory cytokines IL-6 and TNF-α, as it was found that inflammatory conditions can support proteasomal degradation of PDCD4 in macrophages [18].

Besides the regulation of PDCD4 protein levels by degradation, its expression is also controlled by protein biosynthesis. The oncomir miR-21 [37] has been described to be involved in this process by inhibiting translation of *Pdcd4* mRNA [38]. Furthermore, miR-21 is well known to be important for RANKL-driven osteoclastogenesis by downregulation of PDCD4 resulting in AP-1 transactivation and the transcription of osteoclast genes [39]. When we analysed *miR-21* expression (Additional file 2: Figure S2A) we detected a strong upregulation of this miRNA after PMT treatment. However, miR-21 was not rapamycin-dependent.

We therefore propose that PMT-induced miR-21 upregulation is mediated by an alternative pathway of miR-21 induction [40–43] that involves the signal transducer and activator of transcription (STAT)-3, a well-known downstream target of PMT signalling [44] (Additional file 2: Figure S2B). Similar to miR-21 expression, PMT-mediated STAT-3 activation was also rapamycin-independent.

Consequently, transfection of a miR-21 antagomir did not enhance PDCD4 levels after PMT stimulation. This corroborates our data that PDCD4 is regulated via mTOR activation but not via miR-21 (Additional file 2: Figure S2C,D). Data by Sugatani et al. point towards a pivotal role of miR-21 in osteoclastogenesis [39]. Considering that the knock-out of *Pdcd4* does not lead to a osteopenic phenotype in the mouse, miR-21 target genes other than *Pdcd4* play an important role in osteoclastogenesis and are likely targeted by PMT.

Since PDCD4 was described to be central in the transactivation of the AP-1 component c-Jun [19], we investigated whether c-Jun is phosphorylated at its transactivation site Ser63 upon treatment of the cells with PMT (Fig. 4f). We found phosphorylation of Ser 63 already at early time-points (3 h) through a mechanism that appears to be independent of mTORC1. At later time points (24 h) the phosphorylation of c-Jun was dependent on mTORC1. The mTOR-independent, early p-c-Jun molecules (Ser63) might for example be forming heterodimers with other transcriptions factors such as proteins of the ATF, c/EBP, Maf or NF-E2 family driving the transcription of genes not primarily involved in osteoclastogenesis but other cellular process [45]. It also appears plausible that PMT triggers dynamic exchanges of repressive and activating chromatin modifiers (e.g. histone acetyltransferases and deacetylases) associated with AP-1, as these enzymes were shown to control AP-1 dependent gene expression events. Both possibilities require further investigation. At later time points (o.n.), c-Jun is phosphorylated via an mTOR-dependent mechanism and the dimers formed are probably preferentially c-Jun/c-Fos dimers, since these were described before to be crucial for osteoclastogenesis [46, 47].

## Conclusions

In summary, we show that PMT-induced osteoclast differentiation requires mTORC1 activity, which eventually allows the expression of osteoclast specific genes such as cathepsin K via a pathway involving the downregulation of the transcriptional repressor PDCD4. PDCD4 degradation allows JNK to phosphorylate the c-Jun component of the AP-1 complex and transcriptional activation of genes such as cathepsin K. Our data indicate that signalling events related to PMT-induced G protein activation as well as cytokine-induced signals play a role for the activation of mTOR and its role in osteoclast formation.

## Methods

### Reagents

Recombinant PMT and the catalytically inactive mutant PMT^C1165S^ were kindly provided by Dr. Joachim Orth and Prof. Klaus Aktories (Freiburg). Purification of PMT and control of endotoxin contaminations are described elsewhere [24].

### Cell culture

RAW264.7 cells (murine monocytic cell line; ATCC) were cultured in DMEM, supplemented with 10 % heat-inactivated foetal calf serum (Bio West) and 100 U/ml penicillin and 10 μg/ml streptomycin (PAA). The cells were cultivated in cell culture flasks (75 cm^2^; Sarstedt) at humidified atmosphere (95 %), 5 % CO_2_ and 37 °C temperature. The cells were split every 2–3 days in a ratio of 1:30.

### Stimulation

Cells were stimulated with 0.01-5 nM PMT or 50 ng/ml rec. mouse RANKL (R&D Systems).

### Antibodies and inhibitors

Antibodies against p-mTOR (Ser2448), p-p70 S6K1 (Thr389), p-PRAS40 (Thr246), PDCD4, c-Jun, p-c-Jun (Ser63), p-STAT-3 (Tyr705), STAT-3, histonH3, ß-actin were purchased from Cell Signaling Technology. An antibody that recognizes the Q209E modification of Gα_q_ was a gift of Prof. S. Kamitami (Osaka, Japan). Secondary HRP-linked antibodies were obtained from Cell Signaling Technology (anti-rabbit-IgG) or abcam (anti-rat-IgG). The mTOR Inhibitor I (rapamycin) was purchased from Calbiochem and used at 10 ng/ml.

### Quantitative real-time PCR

7.5 × 10^5^ RAW264.7 cells were seeded per well in 6-well plates and then stimulated as indicated. RNA was extracted using High- Pure RNA isolation kit (Roche), according to the manufactures’ protocol. cDNA was prepared by using Revert Aid First strand cDNA synthesis kit (Thermo Scientific). Quantitative RT-PCR was performed using SYBR Green Rox mix (Thermo Scientific) with the primers listed in Table 1. The RT-PCR was run at the 7900 HT Fast Real-Time PCR System (AB Applied Biosystems). An initial denaturation step of 10 min at 95 °C was common for all genes, but the following cycle for annealing and amplification was different. For *Acp5*, *Nfatc1*and *Pdcd4* (40 cycles at 95 °C for 15 s and at 58 °C for 1 min); *Ctsk*, *ATP6v0d2* and *Oscar* (40 cycles at 95 °C for 15 s, 60 °C for 30 s and 72 °C for 30 s). As normalization control *S29* was used. Relative expression (rE) was calculated as rE = 1/(2^ΔCt^). Primers can be found in Table 1.

**Table 1:** Primers used for RT-PCR

Gene	Forward	Reverse
*Acp5*	5´-TTC CAG GAG ACC TTT GAG GA-3´	5´-TTC CAG GAG ACC TTT GAG GA-3´
*Oscar*	5´-AGG GAA ACC TCA TCC GTT TG-3´	5´-GAG CCG GAA ATA AGG CAC AG-3´
*Ctsk*	5´-AGG GAA GCA AGC ACT GGA TA −3´	5´-GCT GGC TGG AAT CAC ATC TT-3´
*Atp6v0d2*	5´-TCA GAT CTC TTC AAG GCT GTG CTG-3´	5´-GTG CCA AAT GAG TTC AGA GTG ATG-3´
*Nfatc1*	5´-GGG TCA GTG TGA CCG AGG AT-3´	5´-GGA AGT CAG AAG TGG GTG GA-3´
*S29*	5´-AGC CGA CTC GTT CCT TTC TC-3´	5´-CGT ATT TGC GGA TCA GAC C-3´
*Pdcd4*	5´-CAC TCA TAC TCT GTT CTT −3´	5´-`TCC ATC TCC TTC ACT TAC −3´

### ELISA

The production of IL-6, TNF-α, IL-12(p40) and IL-10 was measured by commercial assays: mouse IL-6 ELISA MAX™ Standard Set (BioLegend), mouse TNF-α ELISA MAX™ (BioLegend) Standard Set, the BD OptEIA™ Mouse IL-12(p40) ELISA Set (BD Biosciences) or BD OptEIA™ Mouse IL-10 ELISA Set (BD Biosciences). The ELISA assays were conducted according to the protocols of the distributing companies. The TecanGENios Pro plate reader was used to measure the detectable signal. The results were analyzed with the Magellan5 software.

### Western blot analysis

1 x 10^6^ cells were stimulated in 2 ml of medium in a 6-well-format as indicated with or without the inhibitor rapamycin (pre-incubated for 1 h before PMT-stimulation) or solvent control DMSO. Untreated control cells were harvested at the time-point corresponding to the longest time of stimulated cells in this experiment. For the lysis, cells were washed with ice-cold PBS in the well before the cells were scraped of in ice-cold PBS. Cell pellets were resuspended in 100 μl of 1xNP40 buffer (1 % NP-40, 150 mM NaCl, 20 mM Tris (pH 7,4), 10 mM NaF, 1 mM EDTA, 1 mM MgCl_2_, 1 mM Na_3_VO_4_, 10 % glycerin) freshly supplemented with a Phosphatase- and Protease-Inhibitor Cocktail (Roche). Cells were lysed for 40 min at 4 °C and centrifuged to remove cell debris. Lysates were boiled (95 °C; 3 min) in sample loading buffer (50 mM Tris; 2 % SDS, 10 % Glycerol, 100 mM ß-mercaptoethanol) and separated by SDS-PAGE (4-20 % gradient polyacrylamid gel, Anamed). Proteins were transferred to nitrocellulose membrane via semi-dry western blot, blocked in TBST (5 % BSA) for 1 h at RT before the membranes were incubated with the primary antibody, diluted as suggested by Cell Signaling Technology overnight (o.n.) at 4 °C. After 1 h incubation with the secondary antibody (HRP-coupled) (anti-rabbit 1:1000, anti-rat 1:10,000), proteins were detected by enhanced chemiluminescence.

For the detection of p-mTOR (Ser2448), proteins were transferred via a wet-western-blot technique. Lysates were loaded on 7 % SDS-Tris gels and run at 130 V. Proteins were blotted onto a PVDF-Membrane (Roth) at 150 mA for 150 min. Membranes were briefly washed in PBST and then blocked for 1 h in PBST with 5 % milk. Membranes were incubated o.n. in the primary antibody in PBST. Then they were washed three times and then incubated for 1 h with the secondary antibody (Pierce). After washing three times in PBST, membranes were incubated with ECL detection solution (Millipore) and developed.

### Confocal microscopy

RAW264.7 cells were plated on Poly-D-Lysin-coated coverslips (Sigma Aldrich) in 24-well plates at a density of 1 x 10^5^ cells per well in 1 ml of medium. Cells were pre-incubated with rapamycin or corresponding solvent control DMSO for 1 h before cells were stimulated with PMT or RANKL or left untreated. After 4 days of differentiation, cells with enzymatic activity for the enzyme TRAP were fluorescence*-*marked using the ELF 97 phosphatase substrate (Molecular Probes) in an enzyme-labelled fluorescence assay as described by Filgueira et al. [48]. Furthermore the actin filament of the cell was labelled with TRITC-conjugated Phalloidin (Sigma Aldrich) and the nuclei with DAPI (life technology) to distinguish TRAP-positive and multinucleated osteoclasts from undifferentiated RAW264.7 cells. Cells were fixed with 3.7 % paraformaldehyde for 15 min, washed with PBS and permeabilised with 0.2 % TritonX100 for 20 min, washed three times with PBS and incubated with 1 ng/μl TRITC-Phalloidin (PBS; 1 % BSA) for 30 min. Cells were washed three times with PBS and incubated in 50 μl ELF97 phosphatase substrate solution containing tartrate for 13 min, RT. The enzymatic reaction with TRAP was stopped by placing the coverslips for 3 × 4 min on 50 μl stopping solution (Levamisole). After further 3× washing (PBS), cells were incubated with 300 nM DAPI in PBS for 10 min. Cells were washed 3× in PBS, 1x in H_2_O_dd_ before they were mounted.

To visualize the cells, a confocal laser scanning microscope (Leica) was used with a 1.4 NA 63× immersion oil objective. DAPI and ELF97 were excited with UV-light (405 nm) while TRITC was excited with visible light (561 nm). Pictures were taken at 400 Hz with 4× line and frame average.

### Cathepsin K activity assay

RAW264.7 cells were seeded at 1 × 10^5^ cells per well in a 6-well plate. Cells were pre-treated with rapamycin or DMSO for 1 h before cells were stimulated with PMT or left untreated. After 4 days of differentiation, a cathepsin K Activity Assay was performed (Abcam). Cells were lysed in 50 μl of cathepsin K cell lysis buffer and incubated on ice for 10 min. Cell debris were centrifuged at 15,000 rpm for 5 min and the supernatants were removed. The amount of protein in the lysates was determined with a BCA Assay (Pierce) and the amount of protein for each assay was adjusted to 10 μg of protein in 50 μl of lysis buffer per well of a 96-well plate. 50 μl of cathepsin K reaction buffer was added to each well. The Ac-LR-AFC substrate was added to a final concentration of 200 μM and the plate was incubated for 1 h at 37 °C. Fluorescence was measured with a microplate-reader (FLUOstar OPTIMA; BMG LABTECH) with an excitation of 355 nm and an emission of 520 nm.

### Small interfering RNA (siRNA) transfection

PDCD4 and control small interfering RNA (siRNA), transfection medium and transfection reagent were procured from Santa Cruz Biotechnology. siRNA transfection was carried out according to the manufacturer’s protocol. Briefly, 2 × 10^5^ RAW264.7 cells were plated per well in a 6-well plate 24 h prior to transfection. siRNA duplex (60 pmol) was mixed with transfection reagent in transfection medium and added to cells . After 6 h, 1 ml of medium (RPMI 1640 plus 20 % FCS) was added per well; 12 h later, this medium was replaced with standard culture medium (RPMI 1640 plus 10 % FCS). After 24 h, cells were stimulated with PMT or PMT + rapamycin or rapamycin alone for 12 h for qRT-PCR analysis.

### Caspase-Glo 3/7 assay

4 × 10^3^ cells were plated per well in a 96-well plate (clear bottom) and were cultivated in FCS-reduced medium (2.5 % heat-inactivated foetal calf serum) and stimulated with PMT and rapamycin or solvent control DMSO for 48 h (triplicates). Apoptosis was measured using the caspase-Glo® 3/7 Assay (Promega). As positive control, 1 μM doxorubicin (Sigma) was incubated for 6 h before the measurement was performed as described in the manual of the assay provider.

### Fluorescent bone resorption assay

1 × 10^4^ RAW264.7 cells were seeded per well in phenol red free DMEM containing 10 % FBS using a 48-well fluoresceinated calcium phosphate coated plate (Cosmo Bio Co., LTD) as described by manufacturer’s protocol. For differentiation, cells were treated with 5 nM PMT for 6 days, with a change of the medium on every third day. Bone resorption activity was evaluated by measuring the fluorescence intensity at an excitation wavelength of 485 nm and emission wavelength of 590 nm of the conditioned medium as recommended by manufacturer’s protocol using FLUOStar Optima (BMG Labtech).

### Determination of bone resorption pit area

5 × 10^4^ RAW264.7 cells were plated in 1 ml of complete medium in 24-well plates. Cells were either untreated or treated with PMT, rapamycin or in combination of PMT and rapamycin for 3 days. Cells were then transferred to 96 well plate containing bovine cortical bone slices (Boneslices.com) and were treated as mentioned before for 7 days, with a change of the medium on every third day. For measurement of resorbed area, the bovine cortical bone slices were washed with PBS, incubated in 5 % sodium hypochlorite for 1 h, washed twice with water, and stained with 0.1 % toluidine blue. The pits developed a blue to purple colour. The resorbed area was calculated from micro images with Adobe® Photoshop® CS5.

### TRAP staining

5 × 10^4^ RAW264.7 cells were plated in 1 ml of complete medium in 24-well plates. Cells were treated either with RANKL (50 ng/ml) or various concentration of PMT for 3 days. Cells were then fixed and stained using Acid Phosphatase, Leukocyte (TRAP) Kit (Sigma, St Louis, MO, USA). TRAP positive cells with three or more nuclei were scored as osteoclasts.

### Statistical analysis

Data are presented as mean ± SD. Statistical analysis was performed using an unpaired or paired, two-tailed Student’s *t*-test as stated in the corresponding figure legends (*p ≤ 0.05, **p ≤ 0.005,***p ≤ 0.0005) using the Prism 6.0 software (La Jolla, CA,USA).

## Additional files

Additional file 1: Figure S1. SiRNA-mediated knock-down of PDCD4. PDCD4 siRNA transfection was performed according to the manufacturer’s protocol. Transfected cells were lysed 48 h after transfection and β-actin was used as a loading control (*n* = 2). (JPEG 244 kb) Additional file 2: Figure S2. (**a**) PMT-mediated miR-21 activation is mTOR-independent. MiR-21 expression of PMT-stimulated cells was determined by RT-PCR and the effects of mTOR pathway inhibition by rapamycin (10 ng/ml) were addressed (normalisation to *RnU6b*). The indicated standard deviation was calculated from three independent experiments (mean ± SD; *n* = 3). Statistical analysis was performed using an unpaired Student’s *t*-test (****p* ≤ 0.005). (**b**) p-STAT-3 (Tyr705) and STAT-3 was detected by immunoblotting as described above. (**c**) Uptake of the fluorescent 5’6-FAM antagomiR 21 was detected 24 h after transfection by fluorescent microscopy. As controls, untransfected cells and cells transfected with non = fluorescent control antogomiR were used (*n* = 3). (**d**) PDCD4 expression was monitored by western blotting in cells 48 h after transfection with a antagomiR 21 or control antagomiR in the presence or absence of a PMT stimulus (12 h at 5 nM). (JPEG 798 kb)
