# Supplementary figures and images for: Pasteurella multocida toxin- induced osteoclastogenesis requires mTOR activation
Source: Cell Commun Signal. 2015 Sep 14;13:40. doi: 10.1186/s12964-015-0117-7 (PMC4570759; doi:10.1186/s12964-015-0117-7)

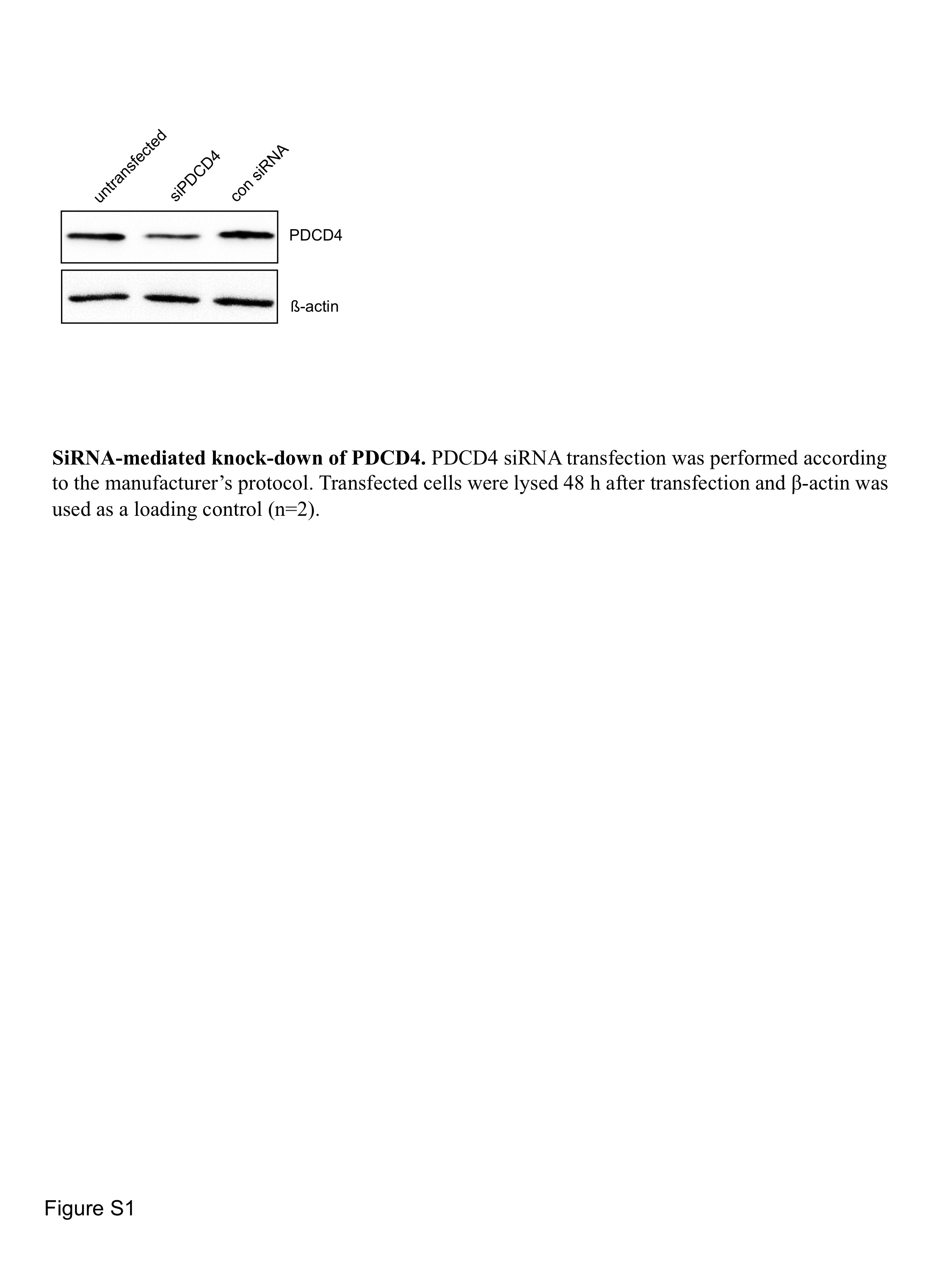

Supplement: Additional file 1: Figure S1. — SiRNA-mediated knock-down of PDCD4. PDCD4 siRNA transfection was performed according to the manufacturer’s protocol. Transfected cells were lysed 48 h after transfection and β-actin was used as a loading control (n = 2). (JPEG 244 kb) [file 12964_2015_117_MOESM1_ESM.jpg]

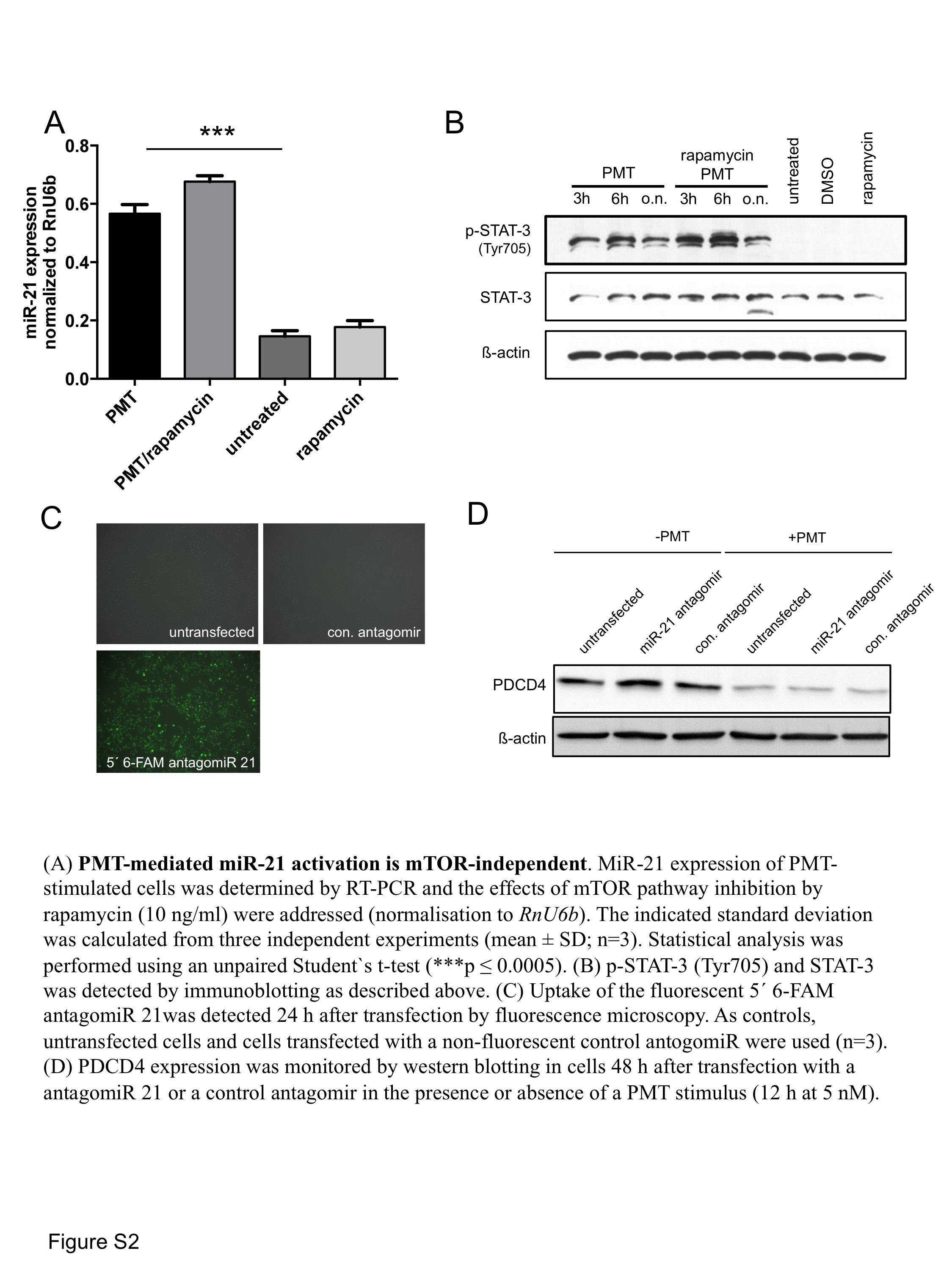

Supplement: Additional file 2: Figure S2. — (a) PMT-mediated miR-21 activation is mTOR-independent. MiR-21 expression of PMT-stimulated cells was determined by RT-PCR and the effects of mTOR pathway inhibition by rapamycin (10 ng/ml) were addressed (normalisation to RnU6b). The indicated standard deviation was calculated from three independent experiments (mean ± SD; n = 3). Statistical analysis was performed using an unpaired Student’s t-test (***p ≤ 0.005). (b) p-STAT-3 (Tyr705) and STAT-3 was detected by immunoblotting as described above. (c) Uptake of the fluorescent 5’6-FAM antagomiR 21 was detected 24 h after transfection by fluorescent microscopy. As controls, untransfected cells and cells transfected with non = fluorescent control antogomiR were used (n = 3). (d) PDCD4 expression was monitored by western blotting in cells 48 h after transfection with a antagomiR 21 or control antagomiR in the presence or absence of a PMT stimulus (12 h at 5 nM). (JPEG 798 kb) [file 12964_2015_117_MOESM2_ESM.jpg]
